# 

**Published:** 1892-09-24

**Authors:** 


### SPECIAL STUDENTS' NUMBER.

CONTENTS.

| | PAGE |
|---|---|
| **The Medicine of the Future** | 417 |
| **To First Year's Men** | 418 |
| **Medical Schools and Degrees** | 419 |
| **List of Hospitals and Medical Schools** | 420 |
| **The Student in Vienna** | 421 |
| **Everybody's Page** | 422 |
| **Annotations.**—Damp Towels—Aristotle's Method of Study—Stands Scotland Where it Did? | 423 |
| **Notes and News** | 424 |
| **Metropolitan and Provincial Medical Schools: Their Cost and Course.** By a PROVINCIAL PHYSICIAN | 426 |
| **Around the Hospitals—Scraps and Gleanings** | 425 |
| **The Practitioner's Mirror and Retrospect—** SURGERY.—On Resection of Intestine | 427 |
| **The Institutional Workshop—** WITHIN THE HOSPITALS.—The Royal Infirmary and Medical School, Newcastle-on-Tyne, 429, The Fleming Memorial Hospital for Sick Children, Newcastle-on-Tyne | 430 |
| HOSPITAL WORTHIES.—Mr. Nixon's Resignation at the London Hospital | 431 |
| **The Cure of Poverty.**—An Account of the North Kensington Friendly Workers among the Poor. By Mrs. C. FURLEY-SMITH | 431 |

**EXTRA SUPPLEMENT.—THE NURSING MIRROR.**

| | |
|---|---|
| **En Passant.**—District Nursing at Camberwell—Clitheroe District Nursing Association—Bathing at San Sebastian—Royal Free Hospital—Another Lady Lecturer—St. Patrick's Home—Nourishment for the Needy—About Hospital Nurses—Midwifery for Nurses and Women Students | clxxxi |
| **Lectures for Asylum Attendants.** By WILLIAM HARDING, M.B. II.—Ventilation | clxxxii |
| **London's End—How to Become a Dispenser** | clxxxiii |
| **Notice to Nurses** | clxxxv |
| **Everybody's Opinion.**—Holiday Homes for Nurses—"Master Mariner" — Belsey v. McLelland — Royal National Pension Fund for Nurses | clxxxiv |
| **Appointment** | clxxxv |
| **Cholera Lectures** | clxxxv |
| **For Reading to the Sick.**—Home | clxxxv |
| **Off Duty.** — "The Result of a Chill." | clxxxvi |
| **Where to Go** | clxxxvi |
| **Notes and Queries—Wants and Workers** | clxxxvi |

